# Medical bioremediation of age-related diseases

**DOI:** 10.1186/1475-2859-8-21

**Published:** 2009-04-09

**Authors:** Jacques M Mathieu, John Schloendorn, Bruce E Rittmann, Pedro JJ Alvarez

**Affiliations:** 1 Dept. of Civil and Environmental Engineering, Rice University, Houston, TX, USA; 2 Dept. of Civil and Environmental Engineering, Arizona State University, Tempe, AZ, USA

## Abstract

Catabolic insufficiency in humans leads to the gradual accumulation of a number of pathogenic compounds associated with age-related diseases, including atherosclerosis, Alzheimer's disease, and macular degeneration. Removal of these compounds is a widely researched therapeutic option, but the use of antibodies and endogenous human enzymes has failed to produce effective treatments, and may pose risks to cellular homeostasis. Another alternative is "medical bioremediation," the use of microbial enzymes to augment missing catabolic functions. The microbial genetic diversity in most natural environments provides a resource that can be mined for enzymes capable of degrading just about any energy-rich organic compound. This review discusses targets for biodegradation, the identification of candidate microbial enzymes, and enzyme-delivery methods.

## Introduction

The continuous renewal of biological components is essential for the proper functioning and survival of mammalian cells. While numerous innate means exist for the elimination of damaged intra- and extra-cellular components, inherent imperfections of these processes inevitably lead to the gradual accumulation of certain compounds. While less of a problem in short-lived or dividing cells, deleterious accumulations may accelerate with increasing age and contribute to the pathogenesis of several major age-related diseases. In fact, it has been proposed that this catabolic insufficiency is one of the principle causes of aging [1-3]. Accordingly, it has been suggested that removal of specific pathogenic compounds that accumulate intracellularly would ameliorate symptoms of certain diseases or aging in general [4,5]. One suggested means of treatment, termed "medical bioremediation," involves the targeted use of exogenous enzymes (or the genes that encode them) to supply missing or enhance insufficient catabolic functions [5]. Medical bioremediation can be considered as an extension of environmental bioremediation, which is "a managed or spontaneous process in which biological, especially microbiological, catalysis acts on pollutant compounds, thereby remedying or eliminating environmental contamination present in water, wastewater, sludge, soil, aquifer material, or gas streams" [6].

Normal turnover of biological structures occurs through numerous pathways. Cytosolic proteins are typically degraded either by the proteosome [7] or a group of calcium-dependent proteases, calpains [8]. Mitochondria utilize Lon matrix proteases [9] and membrane-embedded AAA proteases [10]. Lysosomes, containing a repertoire of hydrolytic enzymes, are capable of degrading a wide variety of macromolecules. A common feature of senescent cells is a reduction in the efficiency of these catabolic pathways [9,11-13]. As a result, cytosolic accumulation of abnormal proteins may occur [12]. The modification of these proteins is thought to be mostly a consequence of post-translational damage that takes place through exposure to reactive oxygen species (ROS) or excess glucose [14]. Another major component of aged cells is lipofuscin, an intralysosomal polymeric material of oxidized protein and lipid found to resist degradation and exocytosis [15]. Oxysterols, generated through oxidative processes within the lysosome [16], may also accumulate intracellularly. Though typically only present at micromolar concentrations, these oxidized derivatives of cholesterol have broad and potent biological activity [17,18]. Extracellularly, amyloid deposits and advanced glycation end-products (AGEs) predominate [19,20]. Although widely diverse in structure and function, each of these compounds disrupts cellular and tissue homeostasis, and accelerates or causes pathogenesis.

The feasibility of medical bioremediation is supported by research showing that proliferating cells may escape accumulation-related senescence through continual dilution, while post-mitotic cells do not. It has also been shown that inhibition of autophagic sequestration or degradation gradually decreased cell viability in confluent fibroblasts, eventually resulting in apoptosis or necrosis [21]. Additional rationale for medical bioremediation comes from the success of enzyme replacement therapy (ERT) in treating lysosomal storage diseases (LSD), a group of inherited disorders resulting from enzymatic deficiency [22-24]. ERT utilizes intravenous injections of exogenous enzyme to replace the non-functional or deficient endogenous enzyme. More recently, preservation of chaperone-mediated autophagy (CMA), which declines with age [25], was reported to restore function in aged mouse livers [26]. This illustrates the possible therapeutic value of removing harmful intracellular aggregates.

This article reviews several major age-related diseases and discusses how they may benefit from medical bioremediation. In addition to detailing how biodegradation of particular aggregates may help prevent these diseases, we provide examples of exogenous enzymes (and the genes than encode them) that may be of therapeutic value. Then, we discuss delivery methods, along with their merits and limitations. Finally, we conclude with a perspective on research needs and potential institutional and commercial barriers to overcome for broad implementation.

## Atherosclerosis

**Figure 1:**
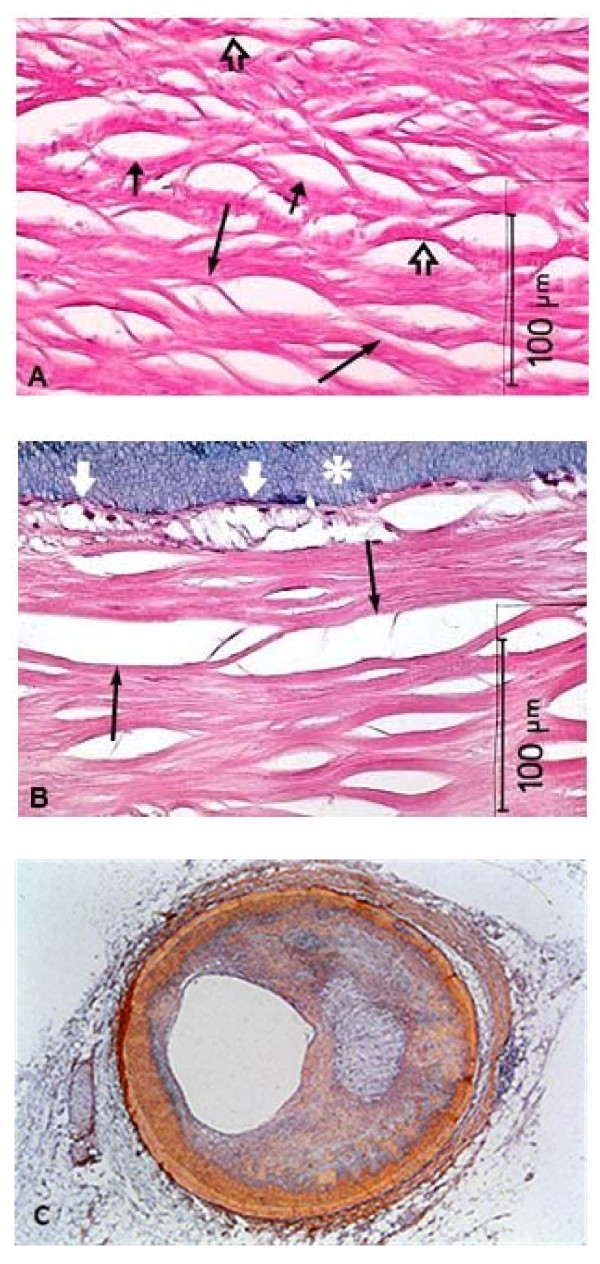
**Coronary heart disease**. **A**, Fibrous plaque from a 65-year old white male showing lipid-laden smooth muscle cells (SMCs) (short arrows). Cells vary greatly in size and some appear to be coalescing (long arrows). The SMC nucleus is flattened against the side of the lacunar space (open arrows). **B**, High-power view of the subendothelial area from **A**. Foam cells (white arrows) have formed beneath the endothelium and lumen (asterisk). Two large, lipid-filled spaces likely formed by the joining of adjacent SMCs that are now dead (black arrows). **C**, Micrograph of a coronary artery cross-section narrowed by a plaque. *Images **A **and **B **courtesy of ncbi.nlm.nih.gov. Image **C **courtesy of genome.gov*.

Atherosclerosis is a progressive disease of the arterial blood vessels and the principle contributor to the pathogenesis of myocardial and cerebral infarction. As such, it is the leading cause of all mortality in the United States, Europe, and Japan [27]. Though the disease is highly ubiquitous, it has an extremely complex etiology that hinders the development of effective treatments. The earliest symptoms are lesions known as "fatty streaks," an aggregation of lipid-rich macrophages and T-lymphocytes within the sub-endothelial matrix that may be a result of arterial injury [28]. Remarkably, these early-stage lesions were found to exist in half of autopsy samples from children aged 10 to 14 [29]. Initiation and progression of "fatty steaks" to fibrous plaques is an inflammatory process that increases cell influx and proliferation at the site of injury, finally leading to the development of the advanced lesions that precede heart attack or stroke (Figure 1).

A major component of atherosclerotic plaques is the foam cell: macrophages or smooth muscle cells containing large amounts of lipid derived from low-density lipoprotein (LDL). LDL is capable of diffusing passively through endothelial cell junctions, and its accumulation is a primary event in atherosclerosis, though its uptake in native form is not rapid enough to generate foam cells [30]. Native LDL, however, is oxidized in the sub-endothelial region [31] or within the lysosome [16] by various processes likely mediated by free radicals or reactive oxygen species (ROS). Extracellular oxidized LDL (oxLDL) undergoes receptor-mediated uptake by macrophage type I or type II class A scavenger receptors (MSR-A) [30], CD36 [32] and lectin-like oxLDL receptor-1 (LOX-1) [33,34]. Unlike the normal route of LDL uptake, these receptor-mediated pathways are not regulated by cellular cholesterol content and may lead to high intracellular levels of oxLDL. Inhibition of MSR-A and CD36 has been shown to reduce atherosclerotic plaque size [35] and foam-cell formation [36]. Conversely, normal macrophages treated with oxLDL quickly become foam cells, accumulating free cholesterol (FC) and displaying reduced lysosomal cholesteryl ester (CE) hydrolysis [37]. OxLDL is now widely regarded as a primary factor contributing to the development of atherosclerotic lesions, having been found cytotoxic to a variety of cell types. OxLDL also possesses a number of other atherogenic properties, such as inhibition of cholesterol efflux, increased expression of cellular adhesion molecules, and stimulation of macrophage proliferation [38-41]. Taken together, these properties may lead to plaque instability, increasing the chance of rupture.

After endocytosis, LDL is delivered to the lysosome, where CE may be hydrolyzed and FC released. Lysosomal FC egress is mediated by Neimann Pick C proteins Type 1 and 2 (NPC) [42], which are believed to transfer FC to acceptor vesicles or directly to the plasma membrane before proceeding to the endoplasmic reticulum [43]. Typically macrophages are protected from excess FC accumulation through acyl-coenzyme A:cholesterol acyltransferase (ACAT) re-esterification in the cytosol and cholesterol efflux. In atherosclerosis, however, intralysosomal FC accumulation occurs, followed by CE accumulation [44]. This series of events has been observed not only in macrophages treated with oxLDL, but in aggregated LDL (aggLDL) and CE dispersion particles (DISP) [45]. Acetylated-LDL (acLDL), however, is not able to achieve FC accumulation, instead forming cytosolic CE inclusions [46]. This is curious, because acLDL is endocytosed by MSR-A, as is oxLDL. AcLDL is not endocytosed by LOX-1, however [33], and was not found to induce apoptosis at the concentration of oxLDL. Similarly, native LDL and cholesterol could not initiate apoptosis at as low of a level as oxLDL [47].

Recent research shows that intra-lysosomal FC accumulation inactivates the vacuolar-ATPases that maintain lysosomal pH, likely by partitioning to the lysosomal membrane and exerting a direct effect on the proteins [37]. This drop in pH subsequently inactivates acid lipase and other hydrolases, leading to CE accumulation. And while it may seem obvious that the unregulated uptake of modified LDL through scavenger receptors could provide the excess FC, the question arises as to why acLDL does not cause the same accumulations, especially considering aggLDL and DISP are unoxidized as well. Answers to this may lie in the resistance of oxLDL to lysosomal degradation [48] and to the observation that acLDL is degraded even more rapidly than native LDL [49]. OxLDL is also more resistant to degradation than either aggLDL or DISP [45], and it is also possible that aggLDL and DISP are oxidized within the lysosome [16].

**Figure 2:**
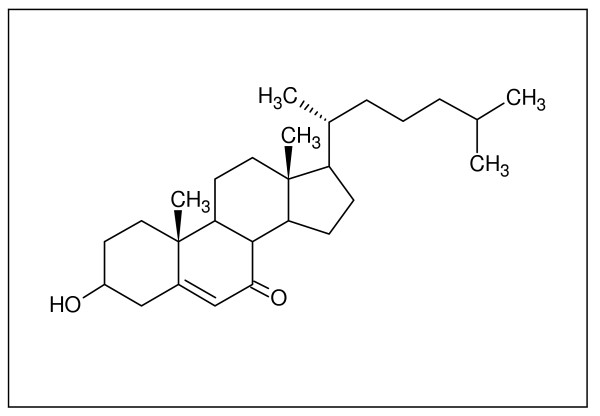
**7-ketocholesterol**. 7KC is an oxidized derivative of cholesterol with cytotoxic properties. It has been associated with atherosclerosis as well as Alzheimer's disease and is thought to destabilize cellular membranes due to its altered physicochemical properties. It is found in high concentrations within atherosclerotic plaques and can be produced from cholesterol by Aβ.

Many of the effects of oxLDL, including cytotoxicity, can be attributed to one of its primary components, 7-ketocholesterol (7KC) (Figure 2) [39]. The average LDL particle contains approximately 600 molecules of cholesterol and 1600 molecules of cholesteryl ester (CE), all of which are susceptible to oxidation prior to and during uptake [50]. Non-enzymatic oxidation of cholesterol predominately occurs at the 7-carbon of the steroid nucleus, a region associated with the greatest cytotoxicity [18,39,40,51,52]. Similar to cholesterol, 7KC partitions intracellularly to the plasma and organellar membranes [53], though its slightly higher polarity alters their biophysical properties [54,55]. Several studies have found 7KC to increase disorder in membrane structure, altering curvature and the properties of nearby membrane-bound proteins [56,57]. Incubation of U937 cells with 7KC caused lysosomal and mitochondrial membrane permeablization in a sequential manner, ultimately inducing either apoptosis or necrosis [51]. Eukaryotic cells contain at least two separate calcium-dependent apoptotic pathways: one modulated by calpain and the other calpain-independent [58]. Calpain has also been implicated in neuronal death following ischaemic insult by initiating lysosomal rupture and subsequent cathepsin B release [59,60]. The ability of 7KC to disrupt cellular Ca^2+ ^homeostasis is likely integral to its toxicity [61-63]. Evidence for this is also supported by the prevention of 7KC-induced mitochondrial damage through the addition of calmodulin inhibitors [64]. How 7KC induces Ca^2+ ^influx is still a matter of research, though it was found that it and several other oxysterols could increase ion conductivity in membranes lacking proteins [65].

Due to its high concentration in atherosclerotic plaques [66-68], cytotoxicity, and other pro-atherogenic properties, 7KC is a prominent target for medical bioremediation. Contributing to the rationale for its elimination is that 7KC has also been associated with Alzheimer's disease by several studies [69-71]. While FC alone has the ability to destabilize lysosomes and hinder CE hydrolysis, the etiology of atherosclerosis is clearly a complex process, and 7KC certainly contributes. Reducing levels of 7KC may subsequently reduce the rate of LDL uptake and apoptosis, slowing atherosclerotic progression.

So how would it be possible to mitigate the effects of 7KC *in vivo*? A number of transformations may reduce its toxicity, with the most obvious example being side-chain hydroxylation. The addition of an oxygen-containing group to the 24- or 27-carbon of the cholesterol side chain increases its ability to traverse cell membranes and allows migration through the blood-brain barrier for easier excretion [72,73]. Sterol 27-hydroxylase catalyzes the formation of 27-hydroxycholesterol, the most abundant oxysterol found in atherosclerotic plaques, and is present in macrophages as well as the liver and several other organs [74-76]. It was also found to utilize 7KC as a substrate, facilitating its secretion [77]. Unfortunately, high levels of 27OH-7KC still accumulate in lesions and foam cells. This may indicate that expression of sterol 27-hydroxylase alone is not sufficient to overcome atherosclerotic progression or that the enzyme is not expressed highly enough to compensate for the increased cholesterol and oxysterol burden.

Another potential route of remediation would be removal of the 7-keto moiety, which generates cholesterol, reducing toxicity to corresponding levels. A mechanism was recently discovered through which 7KC is reduced to 7β-hydroxycholesterol (7β-OH-Ch) by 11β-hydroxysteroid dehydrogenase type 1 [78]. 7β-OH-Ch, however, is more cytotoxic than 7KC; thus, hydrolysis of the hydroxyl group would be necessary. Conversion of 7KC to 7α-OH-Ch is preferable, as the latter has a much reduced toxicity and is normally formed as an intermediate in the production of bile acid in the liver by cholesterol 7α-hydroxylase [79]. Because an enzyme catalyzing the removal of the 7-OH group is not endogenous to humans, this function would have to be supplied exogenously. Our lab recently found evidence of a hydrolase in *Rhodococcus jostii *RHA1 capable of removing a 7-OH group from 7-hydroxycholesterol and a number of its metabolites. Research is being performed to see if this enzyme will be an effective catalyst in human foam cells.

In our studies of *Rhodococcus jostii *RHA1 and in an assay of environmental samples we obtained from soil and activated sludge, we discovered a wide variety of bacteria able to mineralize 7KC [80]. Since RHA1 was known to degrade cholesterol and the pathway previously characterized [81,82], we evaluated its ability to degrade 7KC and performed a transciptomic and metabolite analysis (submitted). As hypothesized, 7KC follows a degradative path similar to cholesterol, though it may utilize multiple isozymes to accommodate the 7-keto substituent prior to its elimination. This pathway involves simultaneous side chain removal and ring cleavage. Humans, however, lack the ability to cleave the ring structure of sterols; so this ability would have to be supplied exogenously. Steroid ring fission may facilitate endogenous enzymatic attack in humans, though this is a purely speculative idea and it is unclear what effect the byproducts would have.

Another potential target for medical bioremediation is CE and oxidized CE. It has been theorized that oxLDL could accentuate accumulation of CE through inactivation of lysosomal acid lipase (LAL). In fact, exogenous supplementation with lysosomal acid lipase was previously shown to reduce the size of atherosclerotic plaques in mice [83].

Though much work remains to be done, the therapeutic value of medical bioremediation in treating atherosclerosis should soon be tested. The broad diversity of organisms displaying the ability to degrade oxysterols provides a wide range of enzymatic mechanisms that can not only effect the transformation of 7KC, but also cholesterol or other sterols that may contribute to the pathogenesis of atherosclerosis.

## Alzheimer's Disease

Alzheimer's disease (AD) is a progressive neurodegenerative disorder that increasingly affects millions worldwide and is the greatest cause of dementia in Western society. It is estimated that US annual incidence rates will surpass 950,000 by 2050, affecting 62% of those 85 and older [84]. It has also been estimated that delaying the onset of AD by just two years would decrease the number of cases in the US in 50 years by approximately two million [85].

**Figure 3:**
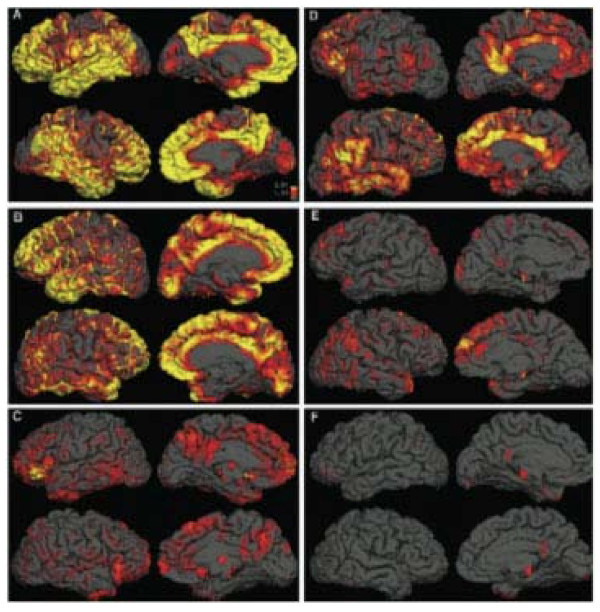
**Amyloid accumulation in Alzheimer's disease**. PET scans reveal Aβ deposits in the brains of three patients with AD (left) and three normal patients (right). Yellow areas indicate high levels of uptake of an Aβ label while red signifies medium uptake. *Image courtesy of lbl.gov*.

AD is characterized by two neuropathological hallmarks: senile plaques composed primarily of extracellular amyloid beta (Aβ) deposits [86,87] and neurofibrillary tangles (NFT) generated from intraneuronal accumulations of abnormal tau protein. Aβ is a peptide of 39–43 amino acids formed by successive cleavage of amyloid precursor protein (APP) by β- and γ-secretases. While Aβ40 is the most common isoform, Aβ42 is most typically associated with progression of AD, accumulating first intracellularly, where it alters the normal metabolism of APP and promotes lysosomal APP accumulation [88] (Figure 3). Tau proteins are microtubule-associated proteins with six isoforms that exist in brain tissue. Hyperphosphorylation of tau causes self-assembly into paired helical filaments that are present in AD and several other tautopathies [89].

The amyloid-cascade hypothesis purports that errors in metabolism of APP are the initiating events in AD pathogenesis, subsequently leading to the aggregation of Aβ (more specifically Aβ42) and eventually plaque formation [19,90,91]. Aβ is thought to catalyze the formation of NFTs [84,92,93], affecting tau proteins through regulation of glycogen synthase kinase-3 (GSK3) activity [94,95] and activation of cyclin-dependent kinase 5 (CDK5) [96]. CDK5 is inactive in its monomeric form and needs p35 for activation, but when cells undergo oxidative injury or treatment with Aβ42, p35 is cleaved by calpain to form p25. This increases the stability of the p25-CDK5 complex, alters its subcellular localization, and eventually results in the hyperphosphorylation of tau [97]. Another relationship between Aβ and tau is their effect on axonal transport, deficiencies of which may promote pathological development. APP is a potential receptor for the kinesin light chain [98], a microtubule motor, and tau has been found to inhibit kinesin-dependent transport [99]. Transport deficiencies have been found to selectively increase Aβ levels and amyloid depositions in the affected regions [100].

The number of relationships among Aβ, tau, and other proteins or disease processes is staggering and beyond the scope of this review; however, it should be noted that many factors involved in AD have also been found determinate of other pathologies. For example, over 100 genes have been found to be associated with AD, and many of these are also highly correlated with atherosclerosis [70,71]. In fact, APP and Aβ can oxidize cholesterol to 7β-hydroxycholesterol, which can be subsequently oxidized to 7KC [101]. Another association between atherosclerosis and AD are the parallels found between NPC and both diseases [102]. Lysosomal dysfunction likely has a primary role in all of these processes [103]. Relationships between frontotemporal dementias, Parkinson's disease, and AD have also been discovered [104]. These may be a result of inflammatory processes, increased tissue transglutaminase [105], or some other factor, but usually the causes of pathogenesis are attributed to just a few compounds. In AD, this is Aβ or tau proteins, both of which may be useful targets for medical bioremediation.

Evidence for the efficacy of eliminating Aβ from senile plaques in the treatment of AD was first reported in mice genetically modified to develop Aβ plaques and subsequently immunized with Aβ42 [106]. This resulted in reduced Aβ plaque formation, astrogliosis, neuritic dystrophy, and improved cognitive function. Aβ42 immunization was able to significantly slow the decline in cognitive function in patients with AD. Unfortunately, post-vaccination meningoencephalitis was seen in a number of patients [107], and follow-up studies determined that Aβ42 clearance did not prevent progressive neurodegeneration [108]. This may be attributed to over-activation of the innate immune system, where a pro-inflammatory response could compromise the potential improvements of plaque reduction. For this reason, plaque reduction using a more transient approach might be valuable in determining the usefulness of Aβ clearance for treatment of AD. It has also been observed that the patients had tau pathology disseminated across the entire cortex [109], implying that elimination of both Aβ and tau may be necessary to achieve a reversal or slowing of disease progression. Animal studies, however, indicate that Aβ immunotherapy is capable of also clearing early stage hyperphosphorylated tau, but not late aggregates [110].

Immunotherapy targeting for hyperphosphorylated tau [111] and α-synuclein [112] has been proven effective for clearing each compound in mouse models of tau pathology and Parkinson's disease respectively. Similar to studies on Aβ immunization [110], the study on tau immunotherapy also revealed that treatment at an early stage was substantially more effective at preserving motor coordination than action taken at later stages of functional impairment. This implies that preventative measures taken before symptoms of AD appear may be most effective. This may well be true for all insoluble aggregates of physiologically soluble proteins.

Aβ and tau clearance may be better achieved using transient expression of enzymes capable of catalyzing their degradation. Additionally, the targeting of intracellular Aβ may overcome some of the limitations of antibody-mediated therapies used to clear extracellular accumulations. Microglia in the CNS were found to impair Aβ-degrading capability, leading to its lysosomal accumulation. Augmentation with mannose-6-phosphorylated hydrolytic enzymes, however, increased Aβ degradation [113]. Although these researchers did not identify the enzymes involved, numerous enzymes already are known to be capable of degrading Aβ. These include neprilysin (NEP) and its homologue endothelin-converting enzyme (ECE1 and ECE2), insulysin (IDE), angiotensin-converting enzyme (ACE), and matrix metalloproteinase-9 (MMP-9). Each of these enzymes has a different subcellular localization, and their use as therapeutics for treatment of AD has already been proposed [114,115]. NEP gene transfer has previously been found to reduce amyloid deposits in transgenic mice [116,117]. Recently, Carty et al. used recombinant adeno-associated virus (rAAV) to over-express ECE in the right anterior cortex and hippocampus of mice, resulting in Aβ reduction [118]. However, expression of these endogenous proteases poses certain risks due to their promiscuous specificity. For example, NEP is known to degrade many substrates with important physiological functions such as enkephalins, substance P, and endorphins [115]. It may be necessary to increase substrate specificity through protein engineering of these particular enzymes to avoid deleterious consequences. Alternatively, proteases with increased substrate specificity may be identified by assaying microbial cultures for the ability to degrade amyloid. In fact, Aβ degradation was recently seen in cell cultures contaminated by *Mycoplasma hyorhinis*. Several other species of mycoplasma are known to be pathogenic and express proteins capable of degrading components of the extracellular matrix. Partial sequencing of the *M. hominis *genome predicted at least three metallopeptidases, and *M. penetrans *is expected to express oligopeptidase O1, a metallopeptidase from the neprilysin family [119]. Some of these enzymes may prove useful tools for remediating Aβ aggregates, either through enzyme replacement or gene therapy.

## Advanced Glycation End-Products

Advanced glycation end-products (AGEs) result from pathways initiated by Maillard reactions between free amines and aldehydic sugars such as glucose. This can result in irreversible glycation and cross-linking of endogenous proteins inside and outside of cells [120]. The first semi-long-lived intermediate of the Maillard reaction is the amadori product called fructosyl lysine. Because of its carbonyl moiety, fructosyl lysine is prone to further attack by primary amines and rearrangement. This gives rise to the heterogeneous mixture of sugar-derived protein adducts summarized as AGEs. Some AGEs are plain adducts to the affected lysine residues, while others may crosslink two or more amino acids from different proteins.

**Figure 4:**
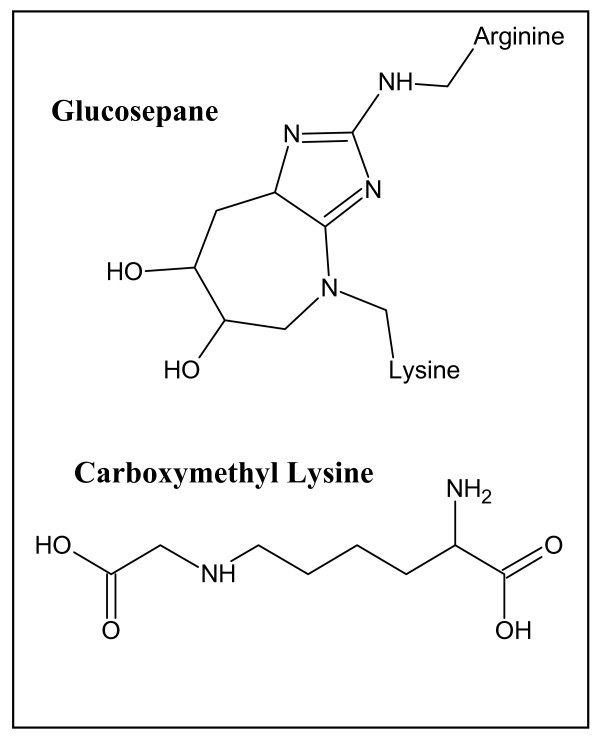
**Advanced glycation end-products**. Two of the most abundant AGEs are glucosepane and carboxymethyl lysine. AGE modification of long-lived proteins contributes to extracellular matrix dysfunction, leading to an array of conditions related to aging and diabetes. These include atherosclerosis, amyloidosis, cataracts, retinopathy, neuropathy, nephropathy, and impaired wound healing.

Among the large variety of AGE structures known, only a few seem quantitatively important enough to have major effects *in vivo *[121] (Figure 4). Glucosepane is by far the most abundant known AGE crosslink [122], and its cleavage is thought to be the most important current objective in AGE remediation [123]. Carboxymethyl lysine (CML) is also of considerable importance, as it has been implicated not only in extracellular matrix dysfunction [124], but has also been suggested to compromise intracellular protein turnover, due to increased glycation of the 26S-proteasome with aging [125]. CML is a non-crosslinked AGE and would, therefore, potentially be the easier target for removal, because access of an enzyme to the AGE may be sterically less impaired, while the chemistry of its cleavage would seem much simpler than in other AGEs.

Proteolytic turnover seems to be a major pathway of AGE removal in human tissues. The resultant free amino-acid AGEs are readily excreted by healthy kidneys and other pathways [126] and are of quantitatively minor importance in comparison to diet-derived free amino-acid AGEs. Thus, AGE modification of most short-lived proteins is thought to be relatively unproblematic. However, some extracellular matrix proteins are so long-lived that significant turnover does not occur within the human life-span. In these proteins (such as certain tissue collagens and lens crystallins), AGEs accumulate with throughout the life span [127]. Because AGEs derive from glucose, they are more abundant and accumulate faster in diabetic patients [128]. AGEs in (long-lived) collagens appear to be better predictors of diabetic complications than those in (short-lived) hemoglobin [129]. Extracellular matrix dysfunction as a consequence of AGE-accumulation is thought to contribute to an array of conditions related to aging and diabetes, including atherosclerosis, amyloidosis, cataracts, retinopathy, neuropathy, nephropathy, and impaired wound healing [130].

AGEs in long-lived proteins are thought to contribute to pathology via two mechanisms. First, by altering the functional groups of amino acids, AGEs may be detrimental to the affected protein's function. In particular, cross-linking of extracellular matrix proteins by AGEs may reduce the mechanical compliance of the tissues, making them more brittle and less able to sustain mechanical stress with age [131]. Second, AGEs are recognized by receptors of AGEs (RAGEs), which modulate inflammatory signaling. Without neutralization of the causative agent, chronic inflammation is thought to promote many age-related diseases [132]. Attempts to attenuate the pathology mediated by AGEs include pharmacological inhibition of the AGE-RAGE interaction, inhibition of their formation, and pharmacological breakage of existing AGEs. Phenylacylthiazolium compounds have been shown to catalytically cleave the α-diketone crosslink, a hypothetical AGE, in vitro [133], and they may have clinical benefits relating to vascular compliance in aged and diabetic patients [134]. However, the α-diketone crosslink has not been demonstrated to occur *in vivo*, and the mechanism of action of this class of compounds remains unclear. No breakers of uncontroversial AGEs *in vivo *are currently known. It appears that the potential of small molecules to specifically catalyze such difficult reactions may be limited.

Because only AGEs in long-lived proteins are thought to play a role in major age-related conditions, a key requirement for any AGE-breaking therapeutic may be the ability to repair AGE-modified proteins, as opposed to merely AGE-modified free amino acids. So far, several examples of AGE-breaking enzymes have been reported that illustrate this requirement. Vincent Monnier et al. [135] began targeting fructosyl lysine, the common precursor to most AGEs, as early as 1994. Using enrichment cultures, they isolated microorganisms that could subsist on fructosyl lysine. They also isolated strains capable of utilizing sterically impaired analogs, such as fructosyl adamantyl amine, as the sole carbon and energy source. This led to the identification of several amine-oxidase type enzymes, called amadoriases, capable of deglycating free fructosyl lysine [135]. However, this class of enzyme does not work on substrates larger than a few amino acids [136]. Furthermore, the amadoriase have resisted extensive attempts of protein engineering to make them work on larger substrates. Recently, detailed structural information has been published, which may explain why this is problem has been so intractable [137].

Van Schaftingen et al. isolated another group of deglycating enzymes from a variety of bacteria as well as humans, the fructosamine kinases. *E. coli *fructosamine-6-kinase (F6K) phosphorylates fructosyl lysine [138], thereby flagging it for cleavage by a subsequent deglycase [139]. However, neither the kinase nor the deglycase have been shown to work on fructosyl lysine modified proteins. Phosphorylation of fructosyl lysine by Fructosamine-3-kinase (F3K) destabilizes the molecule, causing its spontaneous decomposition into lysine, without the need for a deglycase [140]. F3K does work on proteins [141], and this activity appears to constitute a mammalian erythrocyte glycation repair pathway [142]. However, the kinases' ATP requirement is thought to make them unsuitable for extracellular application.

Almost a decade ago, a CML-cleaving enzyme of the amine oxidase family was discovered [143]. However, like its relatives the amadoriases, it is reported that this enzyme does not act on CML-modified protein. It is not yet clear how amenable this enzyme may be to protein engineering to make it work on larger substrates, or whether other CML cleaving enzymes may exist.

No other AGE-cleaving enzymes have been reported. Due to its abundance in collagen, it seems that glucosepane cleavage would be the most important goal for therapeutic application [144]. However, the chemistry involved in cleaving this lysine-arginine crosslink seems highly intractable. At least four bonds must be cleaved to yield the native amino acids, lysine and arginine. Also, access of a cleaving enzyme to the substrate must be even more strongly impaired in the case of glucosepane, because it is a protein-protein crosslink. No attempts to accomplish this have been published.

## Age-related Macular Degeneration

**Figure 5:**
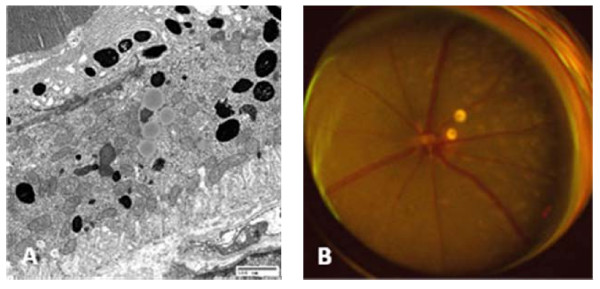
**Age-related macular degeneration**. **A**, A2E deposits in the retinal pigment epithelial (RPE) cells and Bruch's membrane of a senescent CCL2-/- mouse. **B**, Several round, yellow subretinal lesions in the same mouse. *Images courtesy of nei.nih.gov*.

Age-related macular degeneration is the most frequent cause of blindness in the elderly [145]. The disease is characterized by progressive apoptosis of retinal pigment epithelial (RPE) cells, inflammation, extracellular matrix changes, and sometimes aberrant vascularization of the tissue [146]. A large array of pathological mechanisms identified in animal models and human SNP analyses has recently been reviewed [147]. One prominent feature of the disease is the accumulation of pathogenic materials, such as extracellular drusen and intracellular lysosomal lipofuscin (Figure 5).

Drusen are extracellular deposits between the retinal pigment epithelium and Bruch's membrane. Small "hard" drusen are normal features of aging and considered non-pathogenic. In macular degeneration, drusen become larger and diffuse (i.e., "soft"). They contain an array of molecules thought to be actively involved in macular degeneration, such as inflammatory signaling molecules, microglial cells, cell debris, components of the complement system, VEGF, cholesterol, and lipids [148]. These molecules can be modified by protein misfolding, oxidation, and glycation. The heterogeneous nature of the drusen might make it difficult to target these aggregates with catabolic enzymes directly. However, if key modifications could be targeted (such as oxidated or glycated residues), this might render the drusen more amenable to clearance by endogenous mechanisms.

**Figure 6:**
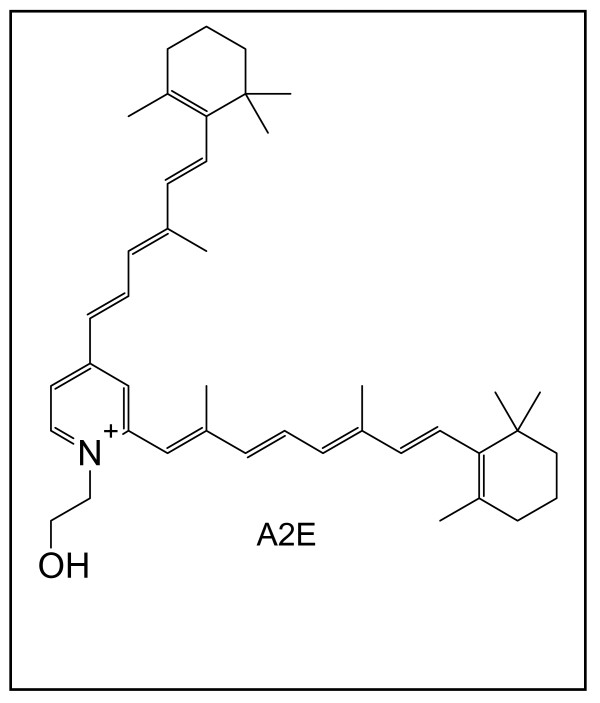
**The pyridinium bisretinoid A2E**. A2E is derived from vitamin A aldehyde (retinal) and phosphatidyl ethanolamine. It accumulates in postmitotic retinal pigment epithelial cells over the life span, and it has been implicated in the ethiology of various forms of macular degeneration.

Lipofuscin is a heterogeneous mixture of indigestible molecules that accumulate in post-mitotic cells [149]. RPE lipofuscin is different from other lipofuscin in that it consists mostly of vitamin-A derived fluorescent compounds, such as the pyridinium bisretinoid A2E (Figure 6). The role of the retinal pigment epithelium is to nourish and support the photoreceptors. A key aspect of this role is the visual cycle, which serves to recycle retinylaldehyde spent in the process of vision. An intermediate of the visual cycle, all-trans retinal, is thought to be the precursor of A2E [150]. And while A2E arises as a by-product of the visual cycle, when retinal reacts with membrane-bound ethanolamine in a rare side-reaction, over the entire life span the total amount of A2E accumulation can be substantial: in aged RPE cells A2E can fill up to 20% of the cell's volume.

A2E has multiple known pathogenic effects. With its hydrophobic side-chains and charged centers, A2E is thought to act as a detergent on membranes. Thus, loading cultured RPE cells with A2E can cause rupture of the plasma membrane, resulting in leakage of cytosolic contents from the cells [151]. Furthermore, the compound can bind to and inhibit lysosomal ATPase, the enzyme responsible for maintaining an acidic pH [152]. The lysosomal pH in cultured RPE cells containing A2E is compromised, possibly to either or both of the above mechanisms [153]. This would likely impair the activity of other lysosomal enzymes, causing broad lysosomal failure. Indeed, the ability of A2E-loaded RPE cells to digest rod outer segments is impaired [154]. Third, A2E is readily transformed into highly reactive species in vivo, by both light and dark mechanisms. Dark mechanisms (autooxidation) yield carbonyls [155]. Light mechanisms (phototoxicity) yield oxiranes [156]. Both types of compounds may react indiscriminately with cellular nucleophiles, including DNA. It is not yet clear which of the above mechanism prevails in causing toxicity, if any. Regardless of the mechanism(s) of toxicity, it seems quite clear that selective enzymatic remediation of A2E would offer a promising new treatment strategy.

The macula is the area of highest photoreceptor density in the retina and serves high-acuity vision. It contains the highest amount of retinol, the highest visual cycle activity, and accumulates A2E the fastest. It is an interesting question whether this may explain why the macula is the first to be affected by age-related degeneration. A2E formation begins by condensation of all-trans retinal with membrane-bound phosphatidyl ethanolamine (PE). It is postulated that the resultant retinlyidene-PE is removed to the outer leaflet by the flippase ABCR (ABCA4, RmP) [157]. According to this model, only retinlyidene-PE that escapes ABCR's flippase activity is available to condense with a second molecule of all-trans retinal to form A2E. This mechanism could be limiting the formation of A2E, and counteracting macular degeneration. ABCR knockout mice, as well as human patients suffering ABCR mutations, accumulate A2E at an accelerated rate and develop early onset macular degeneration (Stargardt's disease) [157,158]. A2E degrading enzymes would help shed light on the role of A2E in heritable and age-related macular degeneration, and might have the potential to become therapeutic agents.

## Gene and Enzyme Discovery

In the previous sections, we provided a number of examples in which the abnormal accumulation of biological "waste" leads to pathological conditions; this situation is further exacerbated by the inefficiencies of cell function found with increasing age. Observations that these accumulations are often slow and progress over the course of a lifetime indicate that the targeted degradation of these deleterious compounds could have enormous therapeutic value if performed before advanced pathology surfaces. In several cases, recombinant human enzymes or antibodies are being tested for their ability to clear these aggregates, but this may upset cellular homeostasis if these enzymes also target unintended substrates or their products are components of regulatory networks. An alternative is the use of xenoenzymes, or enzymes not native to the human cells.

The high levels of diversity found within and among microbial communities provides ample opportunity for the bioprospecting of xenoenzymes capable of transforming any energy-rich carbon-based compound. It has been estimated that 4–6 × 10^30 ^prokaryotic cells exist on earth [159], representing between 10^6 ^to 10^8 ^different taxonomic groups [160]. This is an enormous pool of largely unexploited biological resources. Sifting through this pool for useful enzymes, however, poses certain challenges. Often, the extent of diversity within a given sample can exceed the capacity to effectively screen it. Historically, sample enrichment has been used to overcome this limitation.

**Figure 7:**
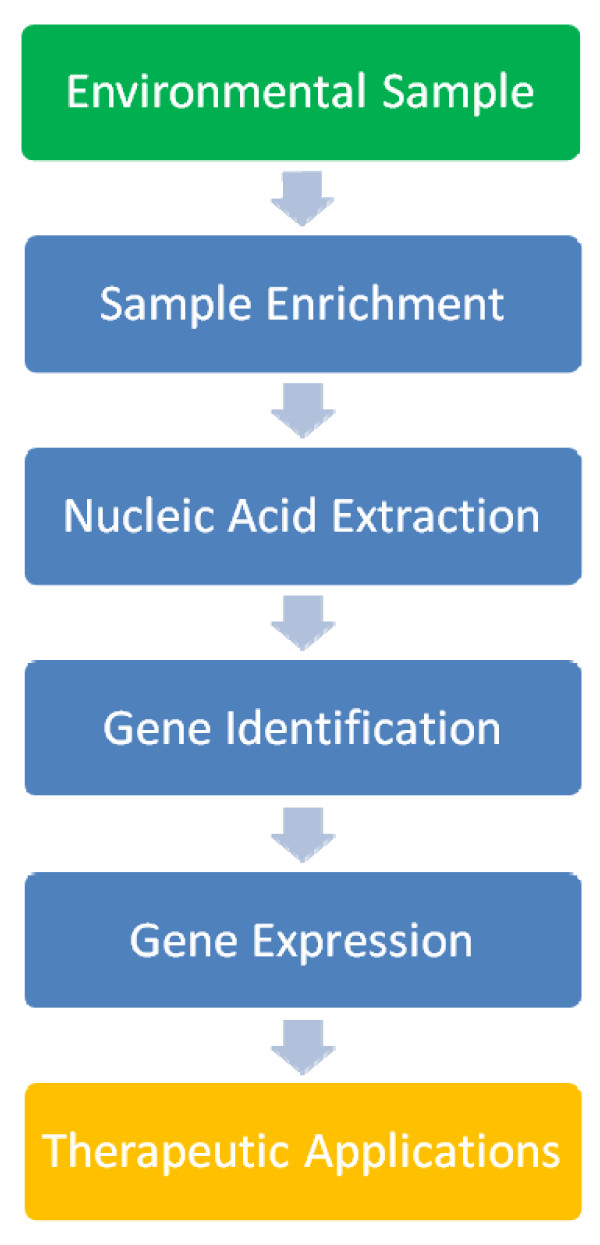
**Gene discovery flowchart**. The search for novel biocatalysts begins with the enrichment of environmental samples. This may be culture, genome, or gene enrichment, and serves to narrow the library size for effective screening. After nucleic acid extraction (DNA or RNA), several avenues are available for gene and enzyme discovery. These can include construction of a metagenomic library followed by functional screening, microarray analysis, or sequence-based assays. Final expression requires full-length gene expression in a heterologous host.

Whole-cell, genome, and gene enrichment are all means of enhancing screening hit rates, improving the discovery of target genes and their corresponding enzymes [161] (Figure 7). Enrichment cultures, involving the use of selective medium for the isolation of one or a small group of organisms, have been used for over a century for the study of biocatalysis, and they help narrow library sizes for effective screening. Our group has used them in conjunction with plating to isolate pure strains of bacteria capable of degrading 7KC [80], and the technique has also been used to identify lipofuscin degraders [162]. Logically, this should be a useful technique for any of the targets we are interested in for medical bioremediation due to the observation that none of these compounds accumulates in the environment. The use of enrichment cultures does have some limitations, however: primarily that current culturing techniques yield a small fraction of the true microbial diversity [160]. Recently, the development of metagenomic techniques [163] has provided a means to harvest the entire genetic complement of environmental samples independent of our ability to culture the microorganisms. These techniques can be used in conjunction with mild enrichment methods to increase screening hit rates, though some loss of diversity will result.

Construction of a metagenomic library relies on effective nucleic acid extraction techniques and cloning directly from environmental samples. Although complete extraction is desired for an even representation of all microbial genomes, this must occur with minimized DNA shearing and as little co-extraction of contaminants as possible. In addition, techniques must be optimized for isolating from different environments [164,165]. Total DNA extraction may also lead to an over-representation of a limited number of dominant microbes. This may be partially resolved using experimental normalization techniques based on cesium-chloride gradient centrifugation in the presence of an intercalating agent, which separates genomes based on their GC content, or methods that require the denaturation and reannealing of genomic DNA. The principle behind the latter method is that abundant ssDNA will re-anneal more quickly, and its extraction will result in an amplification of rare sequences [166].

Genome enrichment often permits the detection and isolation of DNA from metabolically active microbial populations that are not discernable using other techniques. The two most widely used methods of analysis are stable-isotope probing (SIP) and 5-bromo-2-deoxyuridine (BrdU) labeling [167,168]. Genome enrichment by SIP depends on the metabolism of ^13^C-, ^18^O-, or ^15^N-labeled substrates and their utilization for DNA synthesis. The heavier DNA or RNA from organisms catabolizing the substrate can be separated by density gradient centrifugation. Wide application of this technique, however, is limited by the commercial availability of labeled compounds. An alternative is BrdU labeling, which allows the separation of DNA or RNA from community members that are metabolically active, though not necessarily degrading the desired substrate. Label recycling poses problems for both of these methods.

Specific gene enrichment can be achieved by a number of techniques, mostly related to either subtractive hybridization or differential expression. Suppressive subtraction hybridization (SSH) can be used to identify the genetic differences between two bacteria or two complex populations, but does not provide information specific to the genes of interest [169,170]. A more useful technique might be cDNA representational difference analysis (RDA), which relies on mRNA isolation to identify dissimilarities in gene expression. Briefly, cDNA libraries are created using population samples grown under the condition of interest (e.g., in the presence of some substrate) and without the particular condition. These are known as the tester and driver samples, respectively. The derived cDNAs are subjected to digestion and ligated to a synthetic oligonucleotide. PCR is then performed using primers specific to the synthetic oligonucleotides, and the products are digested with the same restriction enzyme. For subsequent steps, the tester is ligated to a different synthetic oligonucleotide and mixed with the driver. Denaturing, annealing, and multiple rounds of PCR with primers specific to the new oligonucleotide amplify sequences unique to the tester in an exponential manner, while other sequences are amplified linearly or not at all [171,172].

The creation and screening of a metagenomic library involves molecular biology methods that have been developed and utilized for over thirty years. One important limitation, however, is achieving gene expression in a surrogate host, which is needed for activity-based screening. The probability of a specific gene being expressed is determined by not only its abundance in the environment, but the insert size, gene length, and expression elements. At a minimum, expression elements must include the *cis*-acting sequences for a promoter and ribosomal binding site (RBS) that are functional in the host. Additional *trans*-acting elements that may need to be present in the host include transcription factors, chaperones, cofactors, or secretory machinery, to name just a few. The *cis*-acting sequences may be engineered into the cloning vector; however, the necessity of *trans*-acting elements is difficult to determine. In looking at options for the incorporation of *cis*-acting sequences, three possible alternatives surface: 1) provision of promoter and RBS from insert, also known as independent gene expression, 2) expression as a fusion using only the RBS from the insert, and 3) expression as a fusion using both promoter and RBS from the vector. Unfortunately, the latter two methods achieve such low frequency of functional constructs that the required number of clones would be too large for most screening assays. Independent gene expression in *E. coli*, while only achieving approximately 40% recovery of enzymatic activity, allows for the creation of much smaller libraries [173]. The use of alternative hosts should allow for greater recovery.

Combining enrichment methods with differential expression technology, such as microarrays, is a powerful method for quickly identifying a large fraction of genes involved in degradation of a particular substrate. In our lab, we were able to identify a *Rhodococcus *sp. capable of degrading 7KC through enrichment cultures. Microarray slides were available for a closely related species, *Rhodococcus jostii *RHA1, and we utilized them for the identification of a number of genes involved in catabolism of 7KC (submitted). Unfortunately, the availability of sequence information is often a prerequisite for microarray slide construction, and when studying a small subset of genes, the prospect of genome sequencing may exceed the time and funding available for a project. One possible solution is the use of a shotgun DNA microarray strategy, which sidesteps the need for sequence information [174,175].

Once an enzyme(s) capable of degrading the target compound has been identified, it must be screened for specificity and the ability to remain active under physiological conditions. Specificity is critical to limit the amount of undesirable catabolism that may potentially disrupt cellular homeostasis. For example, we would like to target 7KC degradation, but cholesterol degradation might not be desirable due to its function in maintaining membrane fluidity. However, considering the number of possible substrates that would have to be screened for each target and the potential lack of commercial availability, *in vivo *assays should be used in conjunction with *in vitro *assays. An exhaustive list of assays would be beyond the scope of this review; however, for protein substrates, which would have the largest diversity space, substrate profiling offers a high-throughput method for screening proteases [176]. Additionally, substrate specificity as well as other operational properties can be modified through protein engineering [177-179].

## Enzyme Drug Delivery

The best way to deliver therapeutic enzymes to human cell lysosomes may be to mimic currently marketed "Enzyme Replacement Therapy" (ERT) drug-delivery routes for heritable lysosomal storage diseases [180-182]. These strategies are based on an affinity interaction of certain "tags" on the enzyme, with a specific endocytic cell surface receptor; binding of the enzyme to the receptor results in endocytosis. According to the classic endocytic route, the endosomes then fuse with lysosomes, resulting in targeted delivery. Thus, enzyme drug delivery to lysosomes is, in our view, mostly a question of which affinity tag/cell surface receptor pairs to use.

In current ERT, two main receptors are used for delivery: the mannose receptor, which is found mostly on macrophages, and the cation-dependent mannose-6-phosphate (M6P)/Insulin-Like Growth Factor 2 receptor (IGF2R), which is found on most cell types [183]. Macrophages are the affected cell types in Gaucher disease, and recombinant glucocerebrosidase is delivered by this route [184]. Other lysosomal-storage diseases, such as Fabry, are diseases that affect most cell types, and the therapeutic is delivered by the M6P/IGF2R route [185].

Using the mannose receptor depends on our ability to manufacture a recombinant therapeutic protein presenting mannose residues at the termini of its glycosylation trees ("mannose-terminated" enzyme). For this purpose, the N-glycosylation apparatus of the endoplasmic reticulum (ER) in eukaryotic cells is usually used. Nascent proteins are synthesized into the ER, while protein folding and glycosylation happen co-translationally. This requires the consensus glycosylation sites "NX(S/T)" to be present on the recombinant protein. If the source of the protein is foreign, or it is not natively glycosylated, then such sites will need to be created on the surface of the protein. Furthermore, NX(S/T) is so short that it may randomly occur or be introduced through point mutations. Thus, internal glycosylation sites will likely need to be removed to permit proper protein folding during the co-translational folding/glycosylation process.

In higher mammalian cells, proteins that receive the core mannose residues in the ER will then be transported to the Golgi apparatus for attachment of higher glycosylations, such as sialic acid [186]. From an ERT point of view, this is undesirable, because the higher sugars will obscure the mannoses and prevent mannose receptor binding.

A few solutions to this problem have been explored. The protein may be treated with glycosidases to remove the higher glycosylations [187]. The protein may be produced in a host that does not use higher glycosylation, such as insect cells [188]. A less well tested possibility may be to use an ER-retention signal (KDEL) to prevent exposure of the recombinant enzyme to golgi-bound higher glycosyl transferase activity.

As the name implies, the mannose-6-phosphate/IGF2 receptor (M6P/IGR2R) is a bifunctional protein – it has distinct binding sites for mannose-6-phosphate and IGF2. It acts as a scavenger for constructs bearing either tag. It is not a signaling receptor, but only responsible for the lysosomal delivery of its substrates for destruction. M6P/IGF2R is also found in the Golgi apparatus, where it directs newly synthesized lysosomal proteins bearing M6P to the lysosome. Due to its natural relevance to lysosomal delivery, traditionally only M6P was used for ERT via M6P/IGF2R. However, M6P attachment sites on lysosomal enzymes are complex and poorly understood. Thus, it is not trivial to attach M6P tags to enzymes that are not natively lysosomal. One way of doing the attachment may be to create a translational fusion of the recombinant therapeutic and a native lysosomal enzyme, in order to "piggy-back" into the lysosome.

A second drawback of using M6P is that the macrophage mannose receptor may internalize M6P-modified protein, clearing it from the bloodstream before it reaches its other cellular destinations[183]. This could be either be due to M6P directly binding to the mannose receptor or to incomplete phosphorylation, leaving some mannose residues intact, which may then bind to mannose receptor. In either case, having an affinity reagent, other than M6P, that would be selective for the M6P/IGF2 receptor may circumvent this problem and enhance delivery to non-macrophage cell types.

One group recently realized that IGF2 as a peptide-tag on their enzyme could achieve lysosomal delivery as well as an M6P tag. This idea was named the glycosylation-independent targeting (GILT) system [189]. Furthermore, IGF2 as a peptide has several advantages over sugars. It can be produced easily and cheaply in a bacterial host. GILT-bearing constructs are not as efficiently cleared from the bloodstream as M6P-bearing constructs, presumably because they do not bind macrophage mannose receptor. This allowed a smaller dose of enzyme to reach its intended target cell lysosomes more efficiently in an animal model [189]. Thus, GILT might *a priori *seem to constitute an acceptable route to deliver xenoenzymes for medical bioremediation to M6P/IGF2R presenting cells.

In summary, it appears that, for the major targets of medical bioremediation – oxysterols, A2E, and AGEs, delivery routes targeting xenoenzymes to their respective destinations are conceivable. The macrophages (oxysterols, atherosclerosis) can be addressed by the mannose receptor. The retinal pigment epithelium (A2E, macular degeneration) has mannose and M6P/IGF2 receptors, and it may be addressable by either or both routes [190]. Those AGEs thought to be most pathologically relevant are extracellular, and they may be accessible without any special enzyme-uptake system.

While ERT may arguably be the most promising form of enzyme delivery for many applications, most lysosomal storage diseases affect the central nervous system (CNS), which is protected by the blood-brain barrier (BBB) [191]. The BBB prevents transport of most chemicals and larger particles from the blood into the brain, while selectively allowing uptake of substances necessary for normal metabolic function. Most enzymes used in ERT cannot cross the BBB; hence, an alternative means of treating the CNS is necessary.

One option is to use the transferrin receptor (TfR) to facilitate transport of therapeutic enzymes across the BBB. This has been achieved in adult mice using monoclonal antibodies (mAb) specific to the TfR conjugated to bacterial β-galactosidase, a 116 kDa protein [192]. Additionally, the TfR was used to deliver plasmid DNA encoding a lysosomal enzyme, β-glucuronidase to the brain. The plasmids were encapsulated in liposomes containing the TfRmAb and intravenous administration was able to bring CNS levels of β-glucuronidase to therapeutic range [193]. Furthermore, these methods were able to delay enzyme clearance in respect to unmodified enzyme due to removal from the blood by the liver and spleen. *Ex vivo *gene therapy has also been used to genetically modify autologous fibroblasts to produce neuronal growth factor (NGF) in the forebrain after grafting [194]. This method could theoretically be used for production of any enzyme in the brain.

Another potential roadblock to the success of ERT is the induction of antibodies to the replacement enzymes by the adaptive arm of the immune system. This is thought to be particularly severe in patients with no detectable levels of the enzyme levels of interest [195] and can greatly reduce the efficacy of treatment. To these patients, the recombinant replacement enzyme appears immunologically foreign. Fortunately, at least three means are available to prevent or attenuate an antigenic response. An immunosuppressive regimen may be useful to reduce or eliminate antibody responses against the recombinant enzyme in many cases [196]. Treatment recipients also may be conditioned for antigen tolerance through regulatory T-cell activation [197,198]. Additionally, proteins may be generated to be immunologically invisible through the incorporation of a specific sequence from Epstein-Barr virus nuclear antigen I that prevents proteosomal degradation of linked antigens [199].

It can be expected that recombinant enzymes from foreign species will face a similar problem. However, when targeting the age-related aggregates discussed above, the situation may be different in several respects. The first major difference is that age-related aggregates accumulate much more slowly than the aggregates in congenital lysosomal storage disorders. For example, AGEs are thought to accumulate only in long-lived proteins, in a process that lasts for the entire life-span [200]. Similarly, lipofuscin accumulations and atherosclerotic lesions grow slowly and in a linear way over the life span. Thus, treatment with the recombinant enzyme may be much less frequent than it is for congenital lysosomal storage disorders. In the ideal case, a highly active enzyme might be administered once in old-age to degrade most of the aggregate present in the body at that time, but before an adaptive immune response has time to develop. The aggregate would then need another life-time to accumulate once again. But even if this ideal case is not achievable, then a transient aggressive immunosuppressive regimen may be used in a controlled hospital environment, where the patient is at little risk for infection for as long as the therapy is required. Due to the age-related nature of the aggregates, it seems unlikely that continuous treatments will be required, where an adaptive immune response would be a major complication.

## Conclusion

The harnessing of microbial catabolic capacity for the treatment of age-related disease offers new therapeutic options for some of the most common maladies of Western society. Since the idea of medical bioremediation was first conceived several years ago [4,5], technical barriers have been overcome, and knowledge has developed that further substantiates its potential feasibility. For example, bacterial enzymes have been expressed in the lysosomes of mammalian cells [201], techniques exist to circumvent the problem of crossing the blood-brain barrier [118,193], and methods of inducing immune tolerance are being actively developed [202-204]. However, widespread implementation of medical bioremediation will depend on the success of trials that will test its efficacy and safety. Our own labs are actively identifying enzymes capable of degrading several important targets; new collaborations and an expanded awareness of this therapeutic option would provide the academic and commercial support necessary to accelerate the development of treatments.

## Competing interests

The authors declare that they have no competing interests.

## Authors' contributions

JM performed the literature review and writing for the introduction, conclusion, and the sections on atherosclerosis, Alzheimer's disease, and gene and enzyme discovery. JS performed the literature review and writing for the sections on age-related macular degeneration, advanced glycation end-products, and enzyme drug delivery. PA and BR provided advice on organizing the manuscript and on editorial quality for all sections. All authors read and approved the final manuscript.
